# Use of Intraoperative Radioguidance in Recurrent Merkel Carcinoma

**DOI:** 10.1155/2020/1789185

**Published:** 2020-06-16

**Authors:** Michèle Beniey, Naomie Destrempes, Geneviève Coulombe, Mona El Khoury, Edgard Nassif

**Affiliations:** ^1^Department of General Surgery, Department of Surgery, Université de Montréal, Montreal, QC, Canada; ^2^Faculty of Medicine, Université de Montréal, Montreal, QC, Canada; ^3^Department of Radiology, Centre Hospitalier de l'Université de Montréal (CHUM), Montreal, QC, Canada; ^4^Department of Surgical Oncology, Centre Hospitalier de l'Université de Montréal (CHUM), Montreal, QC, Canada

## Abstract

Merkel cell carcinoma is a rapidly progressive nonmelanoma skin cancer with a high risk of recurrence. When recurrence occurs, it is associated with poor prognosis and there is a lack of guidelines for the management of such cases. This article describes a challenging case in which the innovative use of iodine-125 radioactive seeds permitted us to precisely identify and resect two nonpalpable recurrent nodules. The safety and accuracy of the surgical procedure were compromised by the presence of scar tissue following two past resections and two courses of radiotherapy. Radioactive seed localization is a well-known procedure in breast cancer, demonstrating potential for an extended application in other cancer types and in complex clinical situations.

## 1. Introduction

Merkel cell carcinoma is a rare and aggressive nonmelanoma skin cancer of neuroendocrine origin [1]. The affected patients are predominantly older Caucasians, and light skin is a risk factor [2]. With a considerable potential for rapid growth, surgery is the mainstay of treatment for the primary tumor. When surgery is performed as the initial treatment, patients tend to have a better overall survival [3, 4]. Patients diagnosed with Merkel cell carcinoma have high recurrence rates of about 48% [3]. The median time to recurrence is about eight to nine months [5]. Recurrences are associated with a particularly poor prognosis [6]. However, there are no specific recommendations for the management of recurrence due to a lack of data in the literature. Treatment is individualized to each patient and mostly consists of surgery and radiotherapy in the context of locoregional disease. Here, we report a case of a recurrent Merkel cell carcinoma extending deeply in the soft tissue of the lower limb. Radioactive localization of two nonpalpable recurring lesions helped to alleviate tumor burden following three progression events.

## 2. Case Presentation

A 71-year-old patient was referred to our surgical oncology department by the medical oncology department for the management of Merkel cell carcinoma. The patient was known for a body mass index of 50, type II diabetes, and stage II colon adenocarcinoma treated with a right hemicolectomy.

Several months ago, the patient had noticed the occurrence of a rapidly progressive nodular lesion on the thigh. A first surgical resection was performed in another center for a 2 cm wide, 22 mm thick Merkel cell carcinoma with a nodular pattern of spread. No sentinel lymph node biopsy had been performed. Both deep and lateral resection margins were positive on pathological analysis. Lymphovascular invasion and intratumoral lymphocytes were found. The mitotic index was 80 mitoses/mm^3^. The tumor was positive for cytokeratin AE1, cytokeratin AE3, keratin 20, and EMA (Epithelial Membrane Antigen).

Two months following local progression, the patient was referred to our institution. We performed a wide local excision together with an inguinal lymphadenectomy. Six out of seven nodes were proven metastatic, and the posterior margin was 0.2 mm close to the tumor. The patient was followed by the medical oncology department, and systemic therapy was not indicated. Later, the patient received a course of adjuvant radiotherapy that was delayed due to impaired wound healing.

During the following year, a PET-CT revealed the presence of a second recurrence in the regional nodal basin. Based on the decision taken during the multidisciplinary cancer conference, the patient received 50 Gy of radiotherapy in the inguinal and para-aortic regions.

**Figure 1:**
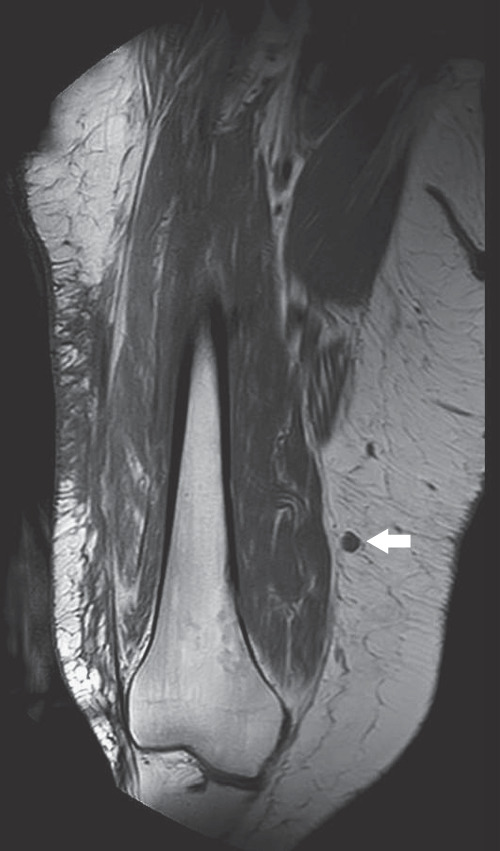
Frontal unenhanced T1-weighted image demonstrating a round deeply located mass in the subcutaneous tissue (arrow) of the lateral aspect of the thigh.

On follow-up imaging, a new 6 mm wide hypermetabolic nodule was identified in the distal thigh (Figure 1), outside of the irradiated field, as well as an in-transit metastasis. The diagnosis of a third recurrence was made. The lesions were not palpable on physical exam.

**Figure 2:**
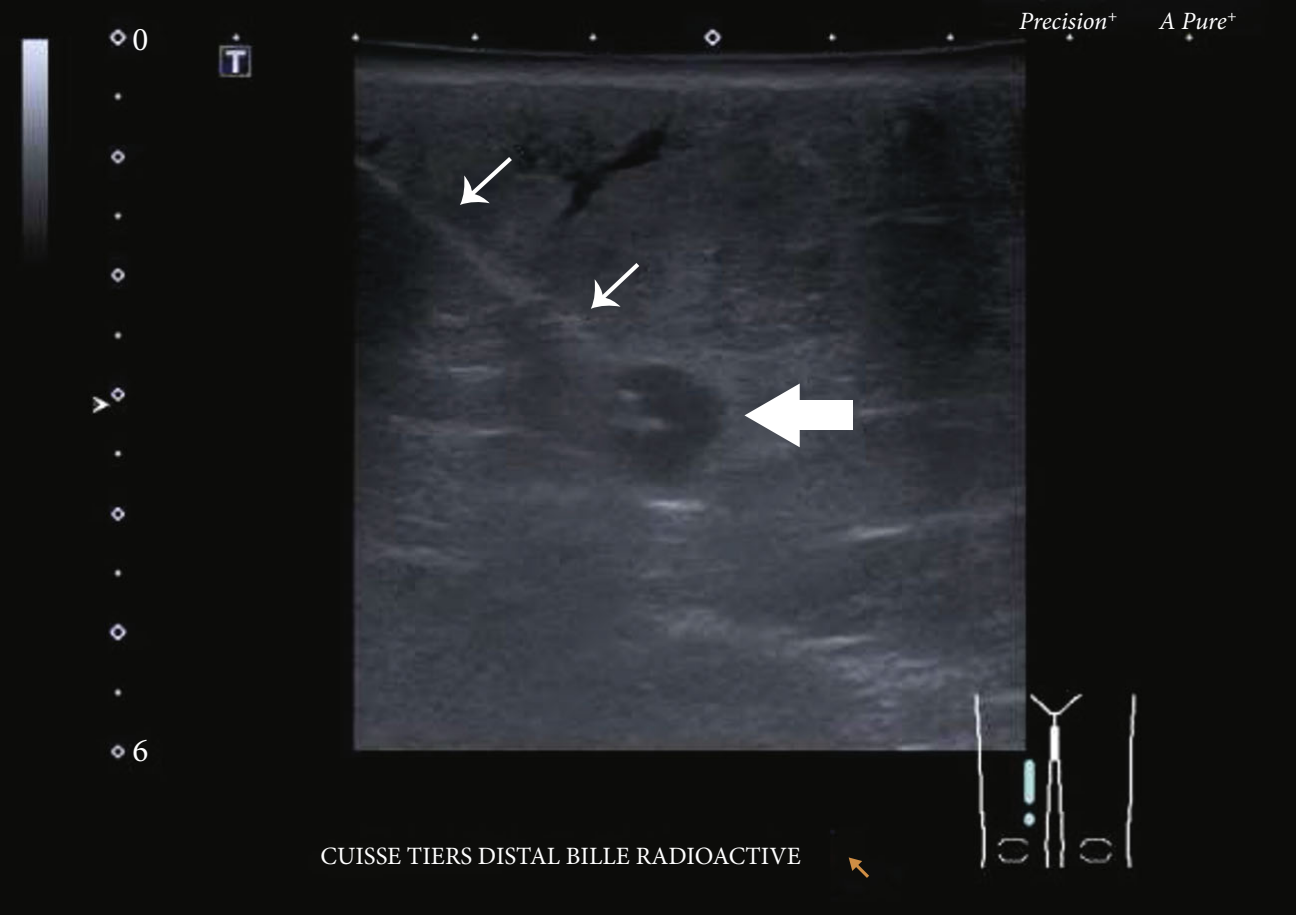
Transverse grayscale ultrasound image. The hypoechoic mass (large arrow) is deeply located in the subcutaneous adipose tissue. The needle (small arrows) is visualized within this mass before deploying the iodine-125 radioactive seed.

**Figure 3:**
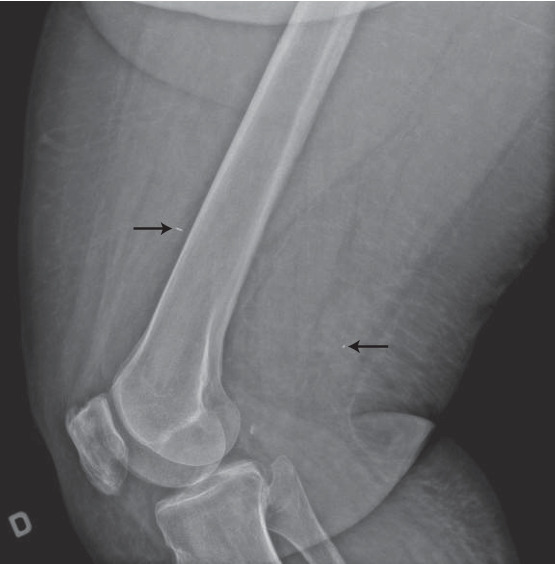
Lateral X-ray of the distal third of the thigh demonstrating the seeds (arrows) projecting on the soft tissue anteriorly and posteriorly to the distal femur.

Considering a significant number of past surgical procedures, the deep localization of the nodule, previous radiotherapy, and a high body mass index, we opted for radioactive seed localization of both lesions. An iodine-125 radioactive seed was inserted under ultrasonography in each nodule (Figure 2), and radiography confirmed adequate positioning (Figure 3). A wide local excision was performed with a radioactive handheld gamma probe, and radiography of the specimen confirmed the successful resection of both lesions. Pathological analysis confirmed the diagnosis of Merkel cell carcinoma. Deep margins were positive, mandating another course of adjuvant radiotherapy. The patient remained free of local and distant recurrence thirteen months following radioactive seed localization.

## 3. Discussion

This article is the first to report on the innovative and judicious use of radioactive seeds to guide the safe identification and dissection of two nonpalpable recurrent Merkel cell carcinoma nodules. In this case report, the radioactive seed localization of a recurrent Merkel cell carcinoma and an in-transit lesion was the most appropriate solution to decrease the risk of an incomplete resection following two surgical procedures. A delay in the administration of adjuvant radiotherapy significantly compromised the oncologic outcome, in the context of positive margins and lymphovascular invasion. The lesions were deep and surrounded by scar tissue from two previous interventions. All these factors can significantly compromise a safe and accurate resection.

We could have also performed an ultrasound-guided resection. However, the use of intraoperative ultrasound is not a well-developed technique and requires the presence of a trained surgeon and a fully equipped operative room.

Radioactive seeds have been initially used in breast-conserving surgery. Indeed, this technique has been used in partial mastectomies with many advantages. Compared to guide wires, radioactive seeds are known for fewer complications such as displacement, and for less discomfort [7, 8]. Further, there is a strong logistical advantage with iodine-125 seeds since seeds are not inserted on the day of surgery. In this case, both radiology and surgery departments can function independently and delays and cancellations in one have a lesser impact on the hospital organization than when guide wires are used.

More recently, several groups have described the use of radioactive seeds to resect proven positive axillary lymph nodes. Targeted axillary dissection is a technique that combines a sentinel lymph node biopsy with the specific resection of a previously proven positive node [9–11]. Patients selected for this procedure have a biopsy-proven metastatic lymph node that completely responded to neoadjuvant chemotherapy clinically. In this subgroup of patients, sentinel lymph node dissection demonstrated higher false-negative rates compared to targeted axillary dissection [9].

Although this technique has been widely used in breast cancer, only few data exist in the literature for skin cancer. Other groups have used radioactive seeds to excise metastatic malignant melanoma [12, 13]. In one case report, a nonpalpable lower extremity recurrent melanoma was retrieved with a radioactive seed, together with an in-transit metastasis [13]. Another group reported using radioactive localization for metastatic melanoma located in the posterior chest wall [12].

As for soft tissue tumors, radioactive seed localization has been described in one study. Garner et al. demonstrated that the iodine-125 seed excision of 10 soft tissue masses resulted in negative margins in nine patients (90%) [14]. One patient with positive margins had a benign tumor and did not require further surgery or treatment [14]. No seed migration occurred, and all seeds were successfully retrieved [14]. Nine out of 10 patients experienced only minimal pain, and all reported a high level of satisfaction [14].

Several intraoperative localization methods have been developed across different cancer types in order to assess resection margins and increase the precision of the resection. First of all, in animal studies, 19 canine and feline sarcomas were analyzed with optical coherence tomography (OCT) to validate tumor resection [15]. In this study, an algorithm was built to process the image in order to obtain better tissue segmentation [15]. The authors found that OCT was able to discriminate between muscle and sarcoma tissues with statistically significant results.

Moreover, a translational study evaluated the use of fluorescence to guide the surgical resection of fibrosarcomas in a murine model [16]. The authors assessed the efficacy of five different agents to detect fibrosarcomas and delineate margins [16]. Luciferase-positive HT1080 cell lines were implanted in 25 mice. More contrast and tumor affinity were seen with cetuximab-IRDye800CW on postadministration day four to nine, with greater tumor fluorescence [16].

In humans, computer-assisted navigation has been essentially used in orthopedic oncology and has the potential to reduce the rate of positive margins following resection [17]. However, only few data resulting from small cohorts are available in the literature and this technique has not been validated in other malignancies [17]. A retrospective review of 24 patients in whom sarcomas were resected with computer navigation revealed low negative margin rates (2/24) [17].

Finally, mass spectroscopy has been utilized to engineer a novel tissue analysis tool [18]. The method was evaluated in 253 patient tissue samples in which an ex vivo molecular analysis was done [18]. Breast, lung, ovary, and thyroid cancer specimens were analyzed [18]. Cancer was detected with a sensitivity of 96.4% and a specificity of 96.2% [18]. More importantly, the classifier was able to detect the presence of a tumor in margins composed of mixed tissue and performed equally in vivo in a breast cancer xenograft model [18]. Although very promising for the resection of deep nonpalpable tissue tumors such as Merkel cell carcinomas, this technique has not been evaluated in this setting.

In conclusion, radioactive seed localization can improve the safety and accuracy of the surgical resection of deep tumors in complex oncology cases. In this patient, the need for a validated intraoperative tumor localization technique was crucial to decrease the risk of incomplete resection. Radioactive seed localization is a promising therapeutic avenue for primary tumor resection in the context of high body mass index and scar tissue due to previous surgery and radiotherapy.

## References

[1] Tothill R., Estall V., Rischin D. (2015). Merkel cell carcinoma: emerging biology, current approaches, and future directions. *American Society of Clinical Oncology Educational Book*.

[2] Albores-Saavedra J., Batich K., Chable-Montero F., Sagy N., Schwartz A. M., Henson D. E. (2010). Merkel cell carcinoma demographics, morphology, and survival based on 3870 cases: a population based study. *Journal of Cutaneous Pathology*.

[3] Liang E., Brower J. V., Rice S. R., Buehler D. G., Saha S., Kimple R. J. (2015). Merkel cell carcinoma analysis of outcomes: a 30-year experience. *PLoS One*.

[4] Wright G. P., Holtzman M. P. (2018). Surgical resection improves median overall survival with marginal improvement in long-term survival when compared with definitive radiotherapy in Merkel cell carcinoma: a propensity score matched analysis of the National Cancer Database. *American Journal of Surgery*.

[5] Santamaria-Barria J. A., Boland G. M., Yeap B. Y., Nardi V., Dias-Santagata D., Cusack J. C. (2013). Merkel cell carcinoma: 30-year experience from a single institution. *Annals of Surgical Oncology*.

[6] Medina-Franco H., Urist M. M., Fiveash J., Heslin M. J., Bland K. I., Beenken S. W. (2001). Multimodality treatment of Merkel cell carcinoma: case series and literature review of 1024 cases. *Annals of Surgical Oncology*.

[7] Bronstein A. D., Kilcoyne R. F., Moe R. E. (1988). Complications of needle localization of foreign bodies and nonpalpable breast lesions. *Archives of Surgery*.

[8] Mittendorf E. A., Caudle A. S., Yang W. (2014). Implementation of the American College of Surgeons Oncology Group Z1071 trial data in clinical practice: is there a way forward for sentinel lymph node dissection in clinically node-positive breast cancer patients treated with neoadjuvant chemotherapy?. *Annals of Surgical Oncology*.

[9] Caudle A. S., Bedrosian I., Milton D. R. (2017). Use of sentinel lymph node dissection after neoadjuvant chemotherapy in patients with node-positive breast cancer at diagnosis: practice patterns of American Society of Breast Surgeons members. *Annals of Surgical Oncology*.

[10] Caudle A. S., Yang W. T., Krishnamurthy S. (2016). Improved axillary evaluation following neoadjuvant therapy for patients with node-positive breast cancer using selective evaluation of clipped nodes: implementation of targeted axillary dissection. *Journal of Clinical Oncology*.

[11] Caudle A. S., Yang W. T., Mittendorf E. A. (2015). Selective surgical localization of axillary lymph nodes containing metastases in patients with breast cancer: a prospective feasibility trial. *JAMA Surgery*.

[12] Dissanayake S., Dissanayake D., Taylor D. B. (2015). Radio-guided occult lesion localisation using iodine 125 seeds "ROLLIS" to guide surgical removal of an impalpable posterior chest wall melanoma metastasis. *Journal of Medical Radiation Sciences*.

[13] Fleming M. D., Pockaj B. A., Hansen A. J., Gray R. J., Patel M. D. (2006). Radioactive seed localization for excision of non-palpable in-transit metastatic melanoma. *Radiology Case Reports*.

[14] Garner H. W., Bestic J. M., Peterson J. J., Attia S., Wessell D. E. (2017). Preoperative radioactive seed localization of nonpalpable soft tissue masses: an established localization technique with a new application. *Skeletal Radiology*.

[15] Mesa K. J., Selmic L. E., Pande P. (2017). Intraoperative optical coherence tomography for soft tissue sarcoma differentiation and margin identification. *Lasers in Surgery and Medicine*.

[16] Prince A. C., McGee A. S., Siegel H., Rosenthal E. L., Behnke N. K., Warram J. M. (2018). Evaluation of fluorescence-guided surgery agents in a murine model of soft tissue fibrosarcoma. *Journal of Surgical Oncology*.

[17] Staats K., Panotopoulos J., Tiefenboeck T. M., Windhager R., Funovics P. T. (2017). Computer navigation-assisted surgery for musculoskeletal tumors: a closer look into the learning curve. *European Journal of Orthopaedic Surgery and Traumatology*.

[18] Zhang J., Rector J., Lin J. Q. (2017). Nondestructive tissue analysis for ex vivo and in vivo cancer diagnosis using a handheld mass spectrometry system. *Science Translational Medicine*.

